# Application of artificial intelligence to quantitative structure–retention relationship calculations in chromatography

**DOI:** 10.1016/j.jpha.2024.101155

**Published:** 2024-11-26

**Authors:** Jingru Xie, Si Chen, Liang Zhao, Xin Dong

**Affiliations:** a School of Medicine, Shanghai University, Shanghai, 200444, China; b Department of Pharmacy, Shanghai Baoshan Luodian Hospital, Baoshan District, Shanghai, 201908, China; c Luodian Clinical Drug Research Center, Institute for Translational Medicine Research, Shanghai University, Shanghai, 200444, China; d Suzhou Innovation Center of Shanghai University, Suzhou, 215000, Jiangsu, China

**Keywords:** Quantitative structure–retention relationship, Chromatography, Accuracy, Machine learning

## Abstract

Quantitative structure–retention relationship (QSRR) is an important tool in chromatography. QSRR examines the correlation between molecular structures and their retention behaviors during chromatographic separation. This approach involves developing models for predicting the retention time (RT) of analytes, thereby accelerating method development and facilitating compound identification. In addition, QSRR can be used to study compound retention mechanisms and support drug screening efforts. This review provides a comprehensive analysis of QSRR workflows and applications, with a special focus on the role of artificial intelligence—an area not thoroughly explored in previous reviews. Moreover, we discuss current limitations in RT prediction and propose promising solutions. Overall, this review offers a fresh perspective on future QSRR research, encouraging the development of innovative strategies that enable the diverse applications of QSRR models in chromatographic analysis.

## Highlights

• AI integration in QSRR models for improved efficiency and accuracy.
• Applications in chromotography, such as compound identification, method development.
• Challenges and potential solutions in QSRR RT prediction.

## Graphical abstract

**Figure:**
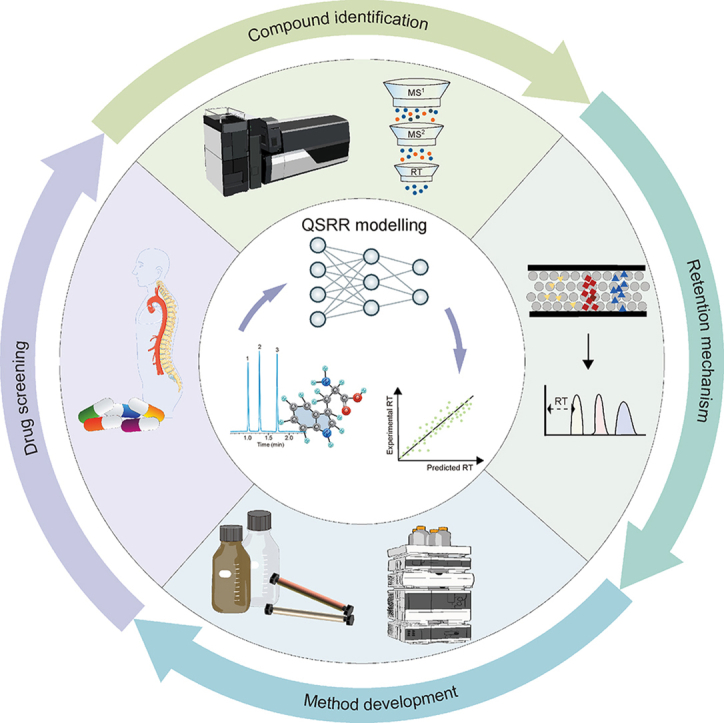

## 1 Introduction

Quantitative structure-retention relationship (QSRR) and quantitative structure-property relationship (QSPR) are subfields of the broader quantitative structure-activity relationship (QSAR) domain [1], which originated in the 1960s and established a foundation for a new scientific field [2]. QSRR, introduced by Kaliszan in 1977 [3], is a valuable tool in chromatography. This approach involves deriving molecular descriptors from the chemical structures of analytes and constructing statistical models to elucidate the relationship between the descriptors and the retention time (RT) of the analytes [4,5]. Chromatography produces extensive datasets of RTs for diverse compounds under uniform experimental conditions, which makes QSRR particularly effective for predicting properties.

RT prediction is widely used in liquid chromatography-mass spectrometry (LC-MS) and gas chromatography-MS (GC-MS) techniques [[6], [7], [8]]. LC-MS is known for its high throughput and precision, making it ideal for analyzing small molecules [9]. GC-MS is particularly effective in untargeted metabolomics, especially for detecting volatile and semivolatile metabolites [8]. LC is categorized into reversed-phase liquid chromatography (RPLC), hydrophilic interaction liquid chromatography (HILIC), and ion chromatography (IC), each with columns designed for specific compound types. RPLC is commonly used for small molecule research, excelling at separating hydrophobic metabolites, whereas HILIC can efficiently separate hydrophilic compounds [6,10]. IC can separate small organic and inorganic ions.

In recent years, artificial intelligence (AI) and machine learning have become essential tools for executing complex tasks across various fields, including chromatography [11]. As early as 1998, AI demonstrated notable advantages in predicting capacity factors, facilitating the development of LC-MS methods [12]. The role of AI in QSRR research has grown substantially owing to its effectiveness in processing complex datasets. This development enhances compound identification, accelerates chromatographic method development, and supports drug screening.

Optimizing the performance of the QSRR model is essential for accurately predicting RT in chromatography. This review highlights the progress of QSRR from an AI-driven perspective and explores the factors that affect model performance and the diverse applications of QSRR in various contexts. Although previous reviews have identified certain challenges in the field of QSRR research, they often lack a comprehensive discussion of strategies to address these challenges. In summary, we outline the major challenges in QSRR research and propose potential solutions, offering researchers a framework to enhance the effectiveness of QSRR in chromatographic analysis and encouraging further innovation in this field.

## 2 Workflow of QSRR

**Fig. 1:**
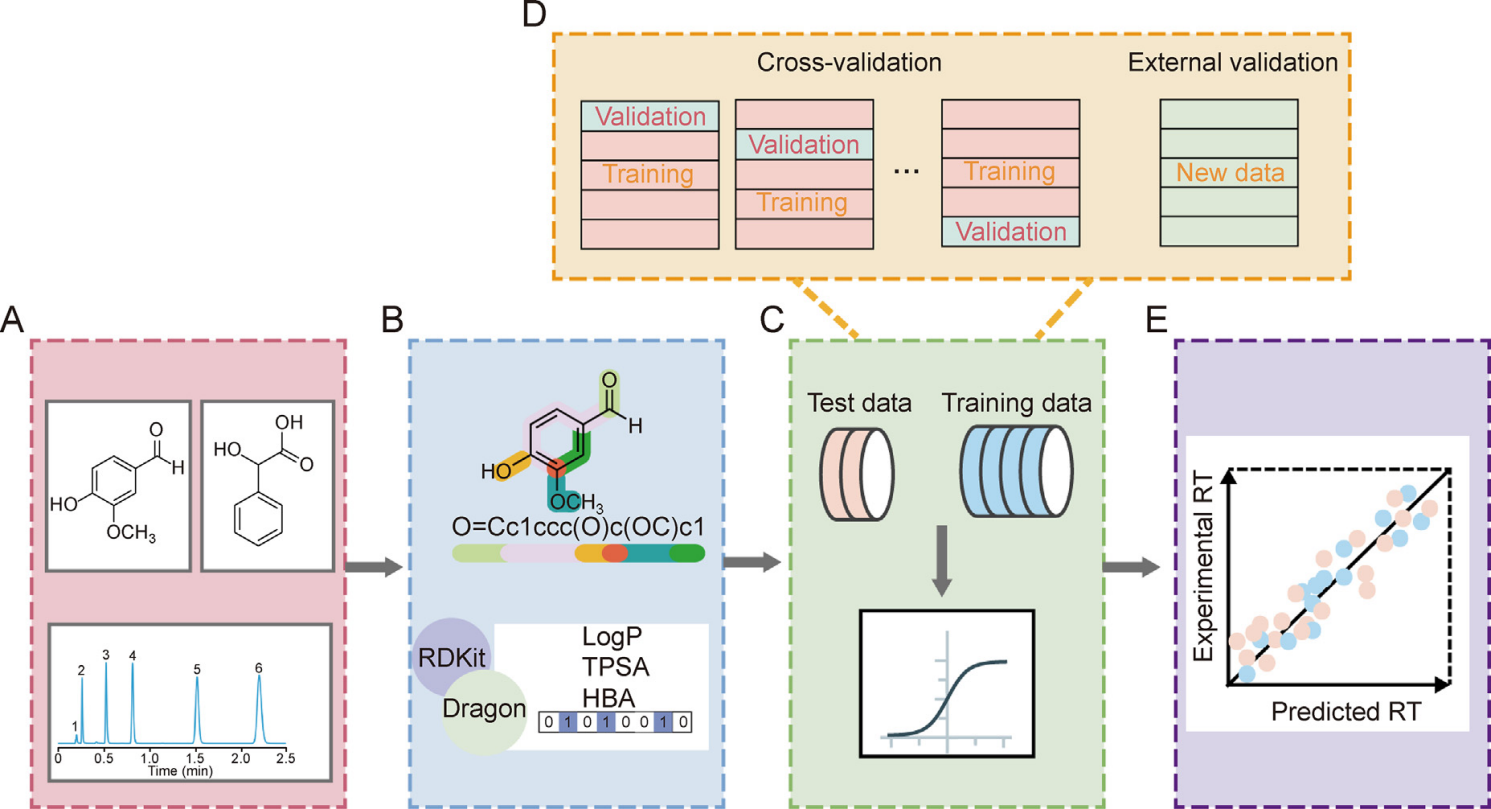
Workflow of the quantitative structure-retention relationship (QSRR) approach. (A) Dataset: Collection of retention times (RTs) and structures of analytes for QSRR modeling. (B) Molecular representation: Calculation and selection of relevant molecular descriptors. (C) QSRR modeling: Data splitting and model development using machine learning algorithms. (D) Model validation: Performance assessment through cross-validation and external validation. (E) Prediction output: RT predictions generated by the constructed QSRR model.

The AI-based QSRR workflow involves the following key steps: 1) obtaining or constructing a database of chemical structures and RTs of analytes; 2) calculating molecular descriptors; 3) selecting appropriate molecular descriptors; 4) splitting the data; 5) building the model using various algorithms; and 6) evaluating the performance of the model. Once validated, the model is used to predict the RTs of target analytes with known chemical structures. The workflow is illustrated in Fig. 1.

### 2.1 Database

In QSRR studies, databases must contain both the RTs and chemical structures of the analytes. The database size significantly affects model performance, with larger databases generally preferred for providing more comprehensive information, thereby enhancing the robustness of the model. Currently, most available databases are in-house collections, typically comprising the data of authentic compounds from commercially available standards and experimental extractions. However, in-house databases often have limited size, which increases the risk of overfitting [13,14]. Small databases are also more susceptible to random correlations, leading to less reliable statistical outcomes and potentially unexpected or inconsistent findings owing to a lack of diversity needed for robust statistical testing [15].

Publicly available databases with RT data have become increasingly valuable. A detailed overview of these databases is provided in Table 1 [14,[16], [17], [18], [19], [20], [21], [22], [23], [24], [25], [26], [27], [28], [29], [30]]. For example, RT data obtained from GC-MS are available in the commercial NIST retention index (RI) [16] and Golm metabolome databases [17]. The METLIN small molecule RT (METLIN SMRT) [14] and in silico Retip datasets [18] provide RT data obtained from RPLC and HILIC analysis, respectively. Because of their extensive size, these datasets are commonly used in deep learning applications and as pretraining resources for transfer learning.

**Table 1:** Common datasets for building quantitative structure-retention relationship (QSRR) models.

Database name	System	Application scene	Refs.
METLIN small molecule retention time database	RPLC	Scenarios that require large amounts of data, such as deep learning, pre-training of transfer learning in RPLC	[14]
NIST Mass spectral/Retention index database	GC	RI prediction of volatile substance	[16]
Golm metabolome database	GC	RI prediction of trimethylsilyl derivatives of metabolites	[17]
In silico Retip database	HILIC	Scenarios that require large amounts of data, such as deep learning, pre-training of transfer learning in HILIC	[18]
Pathogen box database	HILIC/RPLC	Identification of drug candidate compounds exclusively for potential treatment of rare diseases	[18]
Wilson et al. Database	RPLC	Integrating hydrophobic subtraction model and quantitative structure-retention relationship to facilitate the transferability between diverse chromatographicsystems and optimize chromatographic methods	[19,20]
Tan et al. database	RPLC		[21]
PredRet database	HILIC/RPLC	Scenario that needs to transfer RTs between two chromatographic systems	[22]
Fiehn HILIC database	HILIC	RT prediction for small molecules in HILIC	[23]
Hall et al. database	RPLC	RT prediction for small molecules in RPLC	[24]
RIKEN database	RPLC		[25]
Isocratic retention database	RPLC		[26]
Aicheler database	RPLC		[27]
Chiral molecular retention time database	RPLC	RT prediction of chiral molecules and facilitating chromatographic enantioseparation	[28]
Van Laethem database	RPLC	Providing diversity of conditions to support the building of new models	[29]
HighResNPS database	HILIC/RPLC	Identification of new psychoactive substances	[30]

–: no web link was found; RPLC: reversed-phase liquid chromatography; HILIC: hydrophilic interaction liquid chromatography; RT: retention time; GC: gas chromatography; RI: retention index.

A key challenge in predicting RT is the variability in RTs for the same compound across different chromatographic systems, which limits data generalizability beyond the original system. To address this limitation, Wilson et al. [19,20] and Tan and Carr [21] curated two datasets obtained from RPLC analysis and used them for predicting RT by integrating QSRR with the hydrophobic subtraction model (HSM). The partial overlap between these datasets facilitated the evaluation of model performance across different stationary phases. In addition, multisystem RT databases, such as PredRet, have been developed [22] to predict RTs in one chromatographic system based on experimentally determined RTs from another system, improving the transferability of RT data across various systems.

### 2.2 Molecular representation of the analytes

#### 2.2.1 Molecular representation in QSRR

**Fig. 2:**
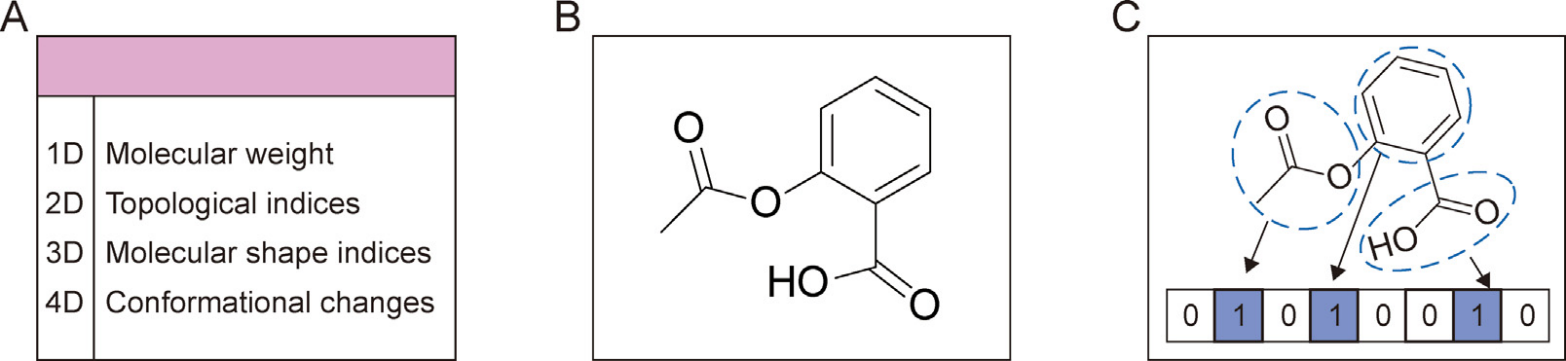
Three types of molecular representation for 2-(acetoxy) benzoic acid. (A) Molecular descriptors: Numerical values that describe various molecular properties across different dimensions. (B) Molecular graph: The chemical structure of the molecule depicted as a graph, where atoms are represented as nodes and bonds as edges. (C) Fingerprint: A binary vector representation of the molecular structure, where the presence or absence of specific substructures or functional groups is encoded as 1s and 0s.

In QSRR research, limited sample sizes and the high dimensionality of features pose challenges. The selection of appropriate molecular representations is crucial for achieving the accuracy and interpretability of QSRR models, making this an important yet complex aspect in this field. Using “QSRR” as a keyword, we searched the SciFinder database, including terms such as “simulation and modeling”, “QSAR”, “QSPR”, “regression analysis”, “molecular structure-property relationship”, and “chromatographic”. After excluding review papers and studies in which QSRR was not defined as quantitative structure-retention relationship, we identified 612 relevant research articles. These studies classified molecular representations into three categories: molecular descriptors, molecular graphs, and fingerprints (Fig. 2).

Molecular descriptors are the most widely used forms of molecular representation in QSRR. The number of molecular descriptors is vast and continuously expanding, as new descriptors are developed to capture various aspects of molecular structures, properties, and activities. These molecular descriptors can be categorized into several types: (1) One-dimensional (1D) descriptors: These are the simplest form of descriptors, relating to the molecular formula, which includes properties such as number of atoms. (2) Two-dimensional (2D) descriptors: These descriptors include topological and connectivity indices and others that consider the relationships between atoms without considering their actual spatial positions. (3) Three-dimensional (3D) descriptors: These descriptors represent the geometric structure of analytes, including steric properties, molecular shape indices, and the distribution of certain chemical groups or features within a molecule. (4) Four-dimensional (4D) descriptors: These descriptors consider the flexibility and conformational changes of molecules over time or under varying conditions. (5) Physicochemical descriptors: These descriptors describe the physical and chemical properties of analytes, including lipophilicity, solubility, and hydrogen bonding potential. (6) Pharmacophore features: These descriptors are the 3D arrangements of features that are important for molecular recognition and binding, such as hydrogen bond acceptors and donors.

**Fig. 3:**
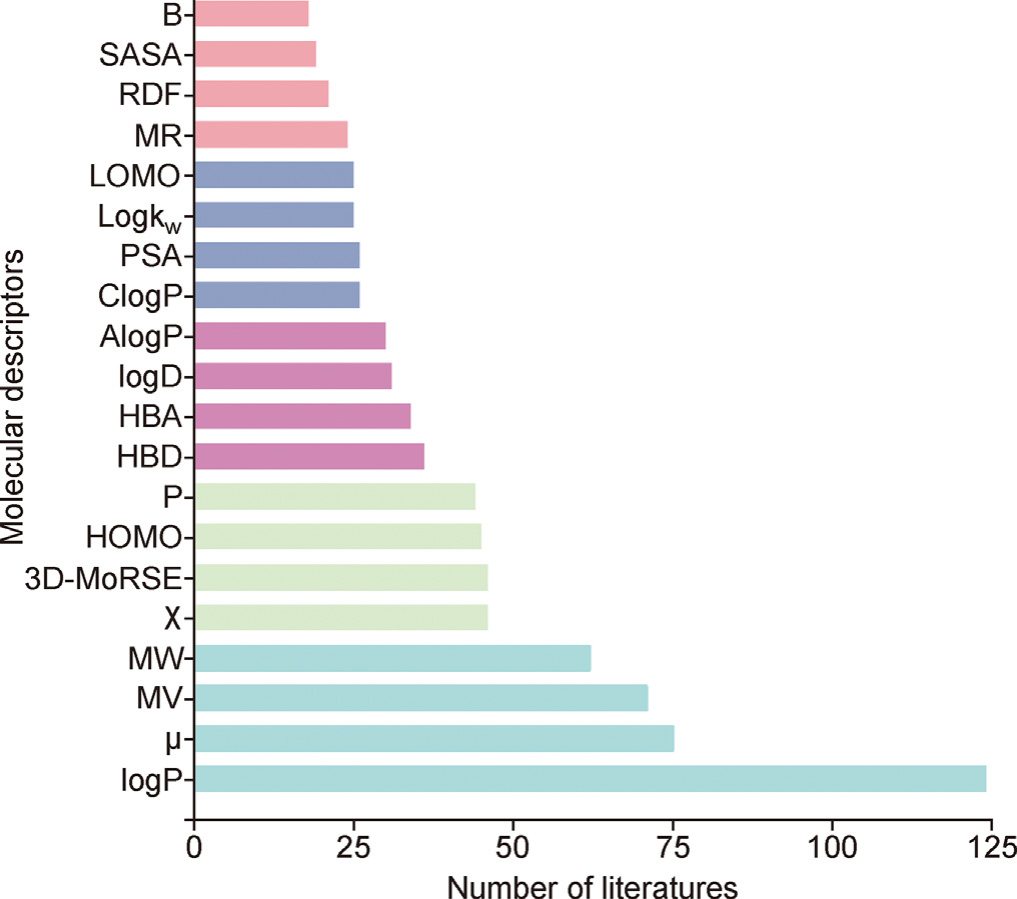
The top 20 most frequently used descriptors in the literature. logP: the octanol-water partition coefficient; μ: dipole moment; MV: molecular volume; MW: molecular weight; χ: connectivity index; 3D-MoRSE: 3D molecular representations of structrue based on electron diffraction descriptors; HOMO: highest occupied unoccupied molecular orbital energies; P: polarizability; HBD: hydrogen bond donors; HBA: hydrogen bond acceptors; logD: distribution coefficient; AlogP: atom-based logP; ClogP: calculated logP; PSA: polar surface areas; logk_w_: the logarithm of the ionization constant; LOMO: lowest occupied unoccupied molecular orbital energies; MR: molar refractivity; RDF: radial distribution function; SASA: solvent accessible surface areas; B: hydrogen bond basicity.

From the 612 QSRR-related research papers, we analyzed the molecular descriptors used to construct QSRR models and identified the most frequently cited descriptors (Fig. 3). Descriptors such as logP, logD, logk_w_, AlogP, and ClogP measure lipophilicity or hydrophilicity, differing in their calculation methods and considering the ionization state of a molecule. These descriptors have been widely recognized for their effectiveness in predicting RTs in chromatographic studies [31,32].

Descriptors related to compound size and shape, such as MV, MW, MR, RDF, SASA, PSA, and 3D-MoRSE, are commonly used to construct QSRR models. These descriptors provide crucial information about the spatial and volumetric properties of compounds, which significantly influence their interactions with the stationary phase during chromatographic separation. The steric factors that affect RT and separation efficiency can be used to enhance our understanding of chromatographic behavior. In addition, their relative ease of computation and interpretation makes them practical tools for developing robust and predictive QSRR models.

Descriptors related to molecular interactions, such as HBD, HBA, and B, play key roles in chromatographic separation. In addition, electronic properties such as the HOMO, LUMO, P, and μ are also important in QSRR studies. The parameter “χ” introduced by Randic in 1975, describes the structure of a compound using a graph-theoretical representation [33]. χ is primarily used to predict the physical, chemical, and biological properties of molecules based on their topological structure, specifically, the connectivity of atoms and bonds, without considering their spatial positions.

Molecular graphs are a popular choice for machine-readable molecular representations in QSRR studies; however, they encounter challenges in accurately representing complex bonds, capturing 3D changes, and addressing computational constraints. In addition, molecular graphs may not adequately represent interactions between molecules and chromatographic systems, which often prompts researchers to prefer simpler or alternative representations in QSRR models. In contrast, fingerprints are valuable features in QSRR modeling that effectively represent analytes and mitigate the common challenge of descriptor selection [34,35]. However, fingerprints typically capture only 2D information and fail to provide geometrical details, resulting in incomplete representations, particularly when dealing with stereoisomers.

#### 2.2.2 Molecular descriptor calculation and selection

The choice of descriptors is a critical aspect of QSRR modeling and must be approached by carefully considering the target problem. Molecular descriptors can be selected based on prior knowledge or derived using computational methods. Many researchers opt for commonly used descriptors guided by empirical insights into retention mechanisms before developing QSRR models [36,37]. For example, the linear solvation energy relationship (LSER) methodology identifies descriptors such as *E*, *S*, *A*, *B*, and *V* through experimental analysis, after which statistical models based on these descriptors are constructed to predict RTs [38]. However, the need for experimental methods to obtain these descriptors limits their applicability.

**Fig. 4:**
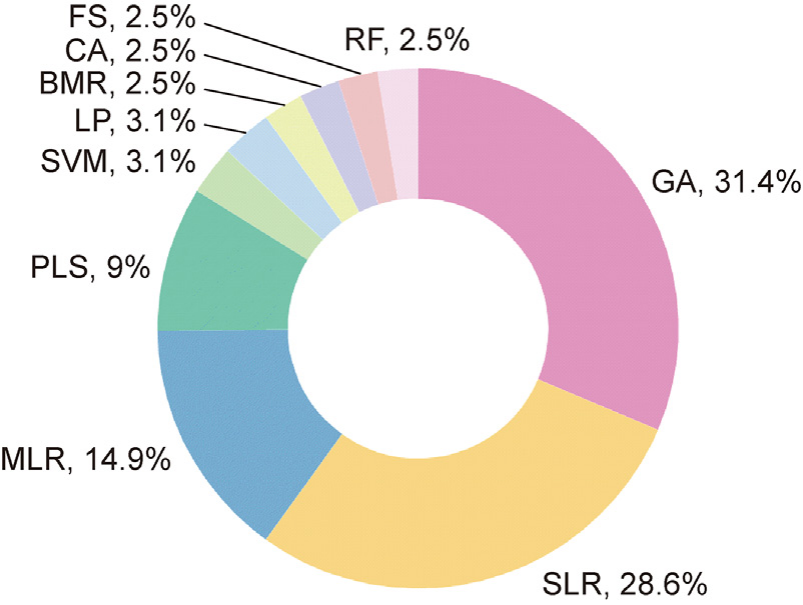
Top 10 most commonly utilized feature selection methods. GA: genetic algorithm; SLR: stepwise linear regression; MLR: multiple linear regression; PLS: partial least squares; LR: linear regression; SVM: support vector machine; BMR: best multilinear regression; CA: correlation analysis; FS: forward selection; RF: random forest.

Various tools, such as Dragon, PaDEL, and RDKit, can calculate descriptors for any given molecule. However, descriptors generated by software often exhibit collinearity and redundancy, which can be considered noise [39]. Therefore, implementing various feature selection methods is essential to identify a suitable subset of features while discarding those that are irrelevant or redundant. This process enhances model accuracy and reduces computation time [40]. An extensive literature review identified 10 common feature selection methods, as shown in Fig. 4. Notably, the genetic algorithm (GA) [41] is the most popular method, and it is frequently combined with regression techniques, such as partial least squares (PLS) and multiple linear regression (MLR), to minimize noise and prevent overfitting [42,43], particularly with large datasets [44]. Although PLS and MLR can address multilinear problems [45], support vector machine (SVM) and random forest (RF) techniques are more commonly applied to develop nonlinear QSRR models although they can also be used for feature selection in specific contexts [46]. The selection of the feature selection method is influenced by the specific analysis requirements and data characteristics. For example, Talebi et al. [47] demonstrated significant improvement in the efficiency and accuracy of QSRR models through feature selection by comprehensively comparing six feature selection algorithms in conjunction with PLS and reference PLS methodologies.

### 2.3 Data splitting and pretreatment techniques for improved QSRR prediction

#### 2.3.1 Data splitting and similarity measures

Data splitting divides a dataset into subsets to validate the performance of the model on unseen data in machine learning [15]. This process is critical for developing accurate models that generalize well and avoid overfitting, and it ensures that the models do not memorize the training data. Methods, such as random and stratified splitting, are employed to ensure that the process is unbiased and representative of the entire dataset. The literature highlights the significant effect of data splitting on the development of QSRR models, particularly when dealing with a small test set [48]. Various algorithms, including the Kennard–Stone algorithm [49], duplex algorithm [50], D-optimal design [50], and K-Artificial neural network (ANN) [51], are used for data splitting. These algorithms focus on selecting data subsets that ensure statistical representation and diversity, thereby facilitating effective model training and validation.

In recent years, many studies have demonstrated that greater similarity between datasets is associated with improved performance of fitted models. Consequently, various similarity metrics have been employed, including the Tanimoto similarity index [52], interactions with stationary and mobile phases [53], LogD [37,54], and retention factor (k) [55]. An overview of these similarity measures is presented in Table 2 [37,[54], [55], [56], [57], [58]]. The Tanimoto similarity index is the most widely used method for assessing the similarity of chemical compounds based on their binary molecular fingerprints [55]. The index ranges from 0 to 1, with higher values indicating greater similarity. However, in one study, a comparison revealed that a model based on k similarity outperformed a model based on the Tanimoto similarity index [55]. Despite these advantages, the k similarity method is limited by its dependence on the availability of k data. To address these limitations, Taraji et al. [56] implemented a dual-filtering strategy that integrated both Tanimoto similarity and RT similarity searching, which identified the nearest neighbor to the target analyte using a molecular descriptor strongly correlated with RT. This dual-filtering approach was more effective than single Tanimoto similarity filtering, achieving a root mean square error of prediction (RMSEP) of 11.01% across various HILIC columns. In addition, this dual-filtering approach has been successfully applied in other studies [55,59].

**Table 2:** Overview of similarity measures used in research articles.

Similarity measures	Description	Refs.
logD	logD ratio (I, II) = logD_I_/logD_II_	[37]
Chemical nature	Compounds were clustered based on their chemical nature, i.e., acids, bases and neutrals.	[54]
k	k-ratio similarity = kI/k_II_	[55]
Dual filtering	Dual filtering involves using a combination of two similarity searches to improve the model performance, such as combinations of structural similarity searching and retention time similarity searching, combining partition coefficient logP searching with SDI-based searching.	[56]
Tanimoto similarity	Tanimoto similarity (I, II) = $\frac{{\text{FP}}_{Ⅰ}\cdot {\text{FP}}_{Ⅱ}}{{\left\|{\text{FP}}_{Ⅰ}\right\|}^{2}+{\left\|{\text{FP}}_{Ⅱ}\right\|}^{2}-{\text{FP}}_{Ⅰ}\cdot {\text{FP}}_{Ⅱ}}$	[57]
SDI	logα = log (*k*/*k*_EB_) = *η′*H – *σ′*S + *β′*A + *α′*B + *κ′*C	[58]

I, II: different compounds; FP: fingerprint; logD: distribution coefficient (logarithmic scale); α: chromatographic selectivity; k: retention factor; k_EB_: the retention factor of ethylbenzene; SDI: second dominant interaction; H, S, A, B, C: five column coefficients; *η′, σ′, β′, α′, κ'*: five complementary solute coefficients.

Local models based on similar compounds often outperform global models; however, their limited generalizability may restrict practical applications. This challenge has led to the development of a successful approach known as the “Federation of Local Models” [60]. Briefly, specific local models are generated for each target compound using only similar compounds, and these local models are then combined to predict the overall chromatographic behavior. This approach has been successfully applied to IC [55], HILIC [56], and RPLC [54]. Tyteca et al. [55] adopted this federation strategy, using k to measure similarity, and found that models trained on highly similar compounds exhibited significantly lower errors, up to eight times lower, than those trained on all compounds. Park et al. [57] demonstrated that a larger and more uniform dataset can facilitate the generation of a training set with higher similarity, resulting in more accurate models. This conclusion is consistent with the findings of Sheridan et al. [61].

#### 2.3.2 Data pretreatment

In QSRR modeling, sparse RT data resulting from variability in experimental conditions can lead to unreliable predictions, particularly in regions with insufficient data points along the RT axis. This uneven data distribution reduces model accuracy in the sparse regions. To mitigate these issues and enhance data alignment with the assumptions of the model, various data pretreatment methods have been employed. A common approach is data transformation, such as applying a logarithmic transformation to RT or k, which addresses skewness and adjusts the data distribution, which is especially beneficial when data are not normally distributed. For example, Sun et al. [62] used the natural logarithm of the experimentally obtained k as the dependent variable to construct a QSRR model for flavonoids, achieving a correlation coefficient (*R*^2^) of 0.9981 and an RMSEP of 6.82%. In addition, standardization and normalization are often used to account for magnitude differences among various features, ensuring that all features are on a comparable scale. Standardization adjusts each feature to have a mean of 0 and a standard deviation of 1, whereas normalization scales the data to a fixed range, typically 0 to 1 [45,59]. Another effective pretreatment technique is principal component analysis, which reduces dimensionality by capturing the main data patterns, thereby simplifying the dataset while retaining essential information [63].

### 2.4 Regression model construction

**Fig. 5:**
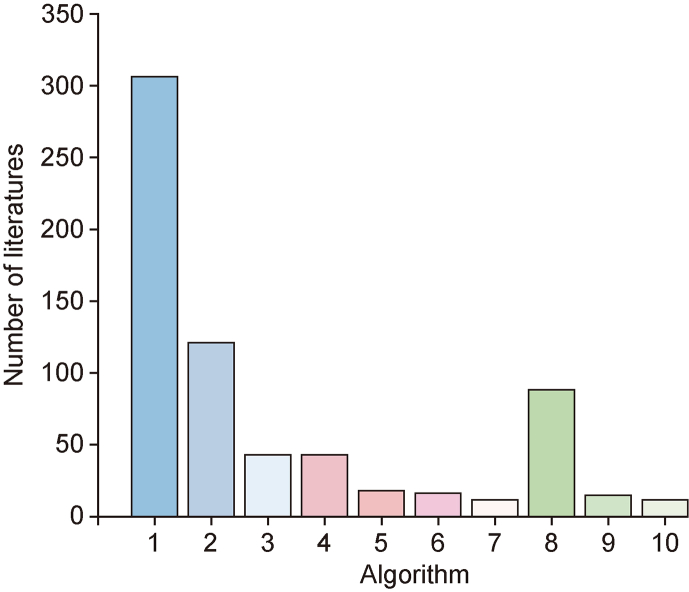
Top 10 algorithms used in literature for constructing quantitative structure–retention relationship model. Number 1, 2, 3, 4, 5, 6, 7, 8, 9 and 10 are multiple linear regression, partial least squares, artificial neural networks, linear regression, support vector machine, random forest, kernel partial least squares, Levenberg-Marquardt artificial neural network, gradient boosting trees and radial basis function neural networks, respectively.

Algorithms are essential for predicting RT because they can accurately model and predict the complex interactions between compounds and chromatographic systems, optimize experimental conditions, minimize the need for experiments, and enhance prediction accuracy. In addition, these algorithms can efficiently process and analyze large datasets of compounds, providing broad applicability and flexibility. The development of RT prediction algorithms dates back to the 1970s, beginning with linear regression, which established correlations between RT data and the connectivity index [3]. However, as instrumental technology rapidly advanced and enabled the collection of diverse data, simple linear regression has become computationally inadequate. This inadequacy has led to the development of more sophisticated regression algorithms. An extensive literature review identified the 10 most common algorithms for constructing QSRR models, as shown in Fig. 5. These regression algorithms can be classified into three main groups: linear regression, nonlinear regression, and neural network algorithms.

#### 2.4.1 Linear regression algorithms

Since the 1970s, linear regression algorithms have been used to predict RT in chromatography. Linear regression generates linear equations that relate descriptors to their corresponding regression coefficients, highlighting the significance of these descriptors and their effect on RT [64]. Ordinary least squares (OLS)-based regression algorithms estimate the parameters in a linear regression model by minimizing the residual sum of squares between the observed and predicted values. MLR commonly uses the OLS method to model relationships involving multiple predictors, making it a popular choice owing to its simplicity and interpretability [65]. However, standard MLR lacks regularization, which can lead to overfitting and instability in the presence of multicollinearity. To enhance these algorithms, regularization terms can be incorporated to address collinearity and improve model performance. Bayesian ridge regression (BRR) [66] and least absolute shrinkage and selection operator (LASSO) regression are examples of such improved algorithms [67]. BRR uses Bayesian inference to estimate coefficients, incorporating prior distributions for regularization and effective handling of multicollinearity. In contrast, LASSO regression adds an L1 regularization term to the loss function, enabling some coefficients to shrink to zero, which facilitates feature selection and enhances model interpretability.

The PLS [68] algorithm addresses multicollinearity by transforming predictors into a smaller set of uncorrelated components. Unlike LASSO and BRR, which adjust parameters continuously, PLS performs dimensionality reduction and regression in separate steps, making it particularly useful for high-dimensional data [69]. This algorithm identifies linear combinations of predictor variables that are maximally correlated with the response variable by iteratively extracting latent variables. These latent variables capture the variance of the predictor variables that are most relevant to the response variables. PLS effectively addresses collinearity among independent variables, particularly when the number of molecular descriptors significantly exceeds the size of the training set.

#### 2.4.2 Nonlinear regression algorithms

Nonlinear regression algorithms are essential for capturing complex data patterns and relationships that linear models cannot adequately represent. In QSRR modeling, several nonlinear regression techniques have been successfully employed, including SVM, decision trees, RF, gradient boosting decision trees (GBT), and kernel partial least squares (KPLS).

Originally proposed for classification tasks, SVM was adapted for regression applications [70]. SVM identifies a hyperplane in the training data that minimizes the distance between the points within the margin and the margin boundary [71]. Different kernel functions, including linear and nonlinear radial basis kernels, can be specified to define the decision function.

Decision trees model the response variable through recursive binary feature division, which separates the response space into homogeneous regions characterized by normally distributed prediction errors. They can handle various data types and scales and are robust to outliers and missing data [72]. Traditional decision trees have two main variations: a bagging version (i.e., RF) and a boosting version (i.e., GBT) [73]. RF combines the predictions of multiple trees by averaging them, which alleviates overfitting [74] and makes it suitable for small sample sizes and high-dimensional feature spaces. In contrast, GBT builds new base learners that maximize their correlation with the negative gradient of the loss function for the entire ensemble [75].

KPLS is a nonlinear, kernel-based algorithm that maps each data point from the original space into a feature space using nonlinear mapping and then constructs a linear PLS model in this transformed space. KPLS avoids the complexities of nonlinear optimization, maintaining the simplicity of traditional PLS while effectively addressing a wide range of nonlinearities using different kernel functions. KPLS outperforms linear models in predicting the RTs of nanoparticles, highlighting its robustness and adaptability [76].

#### 2.4.3 Neural network algorithms

ANNs and their advanced variants, such as deep neural networks and convolutional neural networks (CNNs), possess powerful nonlinear modeling abilities that enable them to uncover hidden features and patterns in complex data [77]. These strengths make them particularly effective at capturing the relationships between molecular properties and RT in QSRR modeling, outperforming traditional linear models and simpler algorithms, especially when working with large datasets [18]. Thus, ANNs have become indispensable and highly effective tools in QSRR research. CNNs, which are known for their success in image recognition, have also been applied to RT prediction. For example, Randazzo et al. [78] used CNNs with 3D electrostatic potential data as input and demonstrated that CNNs outperformed classical algorithms in RT prediction and effectively captured stereochemical information, making them valuable for distinguishing 3D molecular structures. Graph neural networks (GNNs) have garnered attention owing to their strong learning ability on graph data [79]. GNNs leverage information from graph structures to generate low-dimensional, real-valued representations for specific predictions. Yang et al. [80] applied GNNs to direct RT prediction, and they demonstrated that their GNN-RT model outperformed other models, including RF and CNN models. Furthermore, the Levenberg–Marquardt ANN (L-M ANN) and radial basis function neural networks (RBFNNs) are common algorithms used for RT prediction. Shahpar and Esmaeilpoor [81] compared GA-PLS, G-KPLS, and L-M ANN, highlighting the slight superiority of L-M ANN, while Wang et al. [82] demonstrated that RBFNNs outperformed MLR in addressing nonlinear problems for disubstituted benzene derivatives.

#### 2.4.4 Comparison of algorithms

Although significant progress has been made in RT prediction algorithms, two major challenges remain in their evaluation. First, many algorithms are tested on specific datasets, which raises concerns about the generalizability of their reported performance to other datasets. Second, the absence of fair and independent comparisons conducted under consistent conditions complicates the understanding of performance across different scenarios. To improve the evaluation of these algorithms, broader and more objective comparisons are essential. Bouwmeester et al. [83] addressed this issue by comparing seven algorithms across 36 metabolomics datasets using the mean absolute error (MAE), median absolute error, and *R*^2^ as evaluation metrics. The results indicated that no single algorithm outperformed the other algorithms across all datasets, highlighting the need to select an RT prediction algorithm tailored to the specific context.

#### 2.4.5 Impact of different datasets on algorithm performance

The datasets used in QSRR modeling significantly affect the performance of the RT prediction algorithms. Dataset size is a key factor; early algorithms often relied on limited samples or subjective tuning, which restricted their effectiveness [15,84]. However, with the availability of large databases, deep neural network algorithms can now use more diverse datasets, significantly enhancing the accuracy and performance of RT prediction [[85], [86], [87]]. A sufficiently large dataset with various analytes [14] and chromatographic conditions [22] facilitates more comprehensive feature learning, thereby improving prediction accuracy and stability. In addition, dataset characteristics such as the similarity between training and test sets also play a crucial role; greater similarity enables the model to apply learned features more effectively, which results in better overall performance.

#### 2.4.6 Metrics for evaluating model performance

The quality of a model should be evaluated in three dimensions: accuracy, predictability, and predictivity. Early studies often equated a high *R*^2^ value with model accuracy [88]. However, contemporary research has demonstrated that, while a model with strong performance typically exhibits a high *R*^2^ value, this metric alone is insufficient to ensure model quality [89,90]. Therefore, additional evaluation criteria are essential for assessing model accuracy.

For a QSRR model to be suitable for practical application, it must demonstrate a high R^2^ and low error during validation [91]. In a study conducted by Taraji et al. [92], four error-based validation metrics—MAE, RMSEP, MAE scaled to RT (%MAE), and RMSEP scaled to RT (%RMSEP)—were compared to identify the most appropriate criteria for validating QSRR models and assessing their accuracy. The findings indicated that %RMSEP is the most reliable indicator of the predictive ability of a model because it avoids the pitfalls of absolute error, which can be influenced by the RT values of individual analytes, making it less applicable when there are significant differences in RTs. Additionally, %RMSEP places greater emphasis on larger errors by considering the sum of the squares of individual errors, thus providing a more accurate representation of the true predictive ability of the model. The evaluation criteria for QSRR performance are detailed in Table 3.

**Table 3:** Formulas for various metrics to evaluate the accuracy and predictivity of quantitative structure-retention relationship (QSRR) models.

Metrics	Formula
*R*^2^	$R^{2}=1-\sum_{i=1}^{n}{\left(y_{i}-\hat{y_{i}}\right)}^{2}/\sum_{i=1}^{n}{\left(y_{i}-\bar{y}\right)}^{2}$
MAE	$\text{MAE}=\sum_{i=1}^{n}\left|\left(y^{i}-\hat{y}\right)\right|/n$
RMSEP	$\text{RMSEP}=\sqrt{\frac{\sum_{i=1}^{n}{\left(y^{i}-\hat{y}\right)}^{2}}{n}}$
RMSECV	$\text{RMSECV}=\sqrt{\frac{\sum_{i=1}^{n}{\left(y^{i}-\hat{y}\right)}^{2}}{n}}$
%MAE	%MAE = $\frac{\sum_{i=1}^{n}\frac{\left|\left(y^{i}-\hat{y}\right)\right|}{y^{i}}}{n}$ ∗100
%RMSEP	%RMSEP = $\sqrt{\frac{\sum_{i=1}^{n}{\left(\frac{y^{i}-\hat{y}}{y^{i}}\right)}^{2}}{n}*100}$
Q^2^_ext_	$Q^{2}$_ext_ = 1–$\sqrt{\frac{\sum_{i=1}^{n}{\left(y^{i}-\hat{y}\right)}^{2}}{\sum_{i=1}^{n}{\left(y^{i}-\bar{y}\right)}^{2}}}$
MedAE	$\text{MedAE}=\text{median}\left(\left|y^{i}-\hat{y}\right|\right)$

*R*^2^: correlation coefficient; n: the number of compounds; $y^{i}$: actual retention time, $\hat{y}$: predicted retention time, $\bar{y}$: the average of the observed retention time of the target analytes; n: the number of test analytes; MAE: mean absolute error; RMSEP: root mean square error of prediction; RMSECV: root mean squared error of cross-validation; %MAE: MAE scaled to retention time; %RMSEP: RMSEP scaled to retention time; Q^2^_ext_: external validated coeffificient of determination; MedAE: median absolute error.

Model predictability is closely associated with its applicability domain (AD), which depends on the diversity of compounds in the training set and the molecular descriptors selected [93]. The AD can be visualized through a Williams plot [51] with a model featuring broader ADs that typically show improved predictability. However, no model trained on a specific training set may perform well when predicting the RTs of different analyte types.

Model predictivity is essential [91] for assessing the robustness of a model and is typically evaluated using two primary methods: cross-validation and external validation [15,93]. In QSRR, common cross-validation techniques include leave-one-out (LOO) and leave-more-out (LMO). LOO iteratively removes one sample from the training set at a time to construct a model that predicts the dependent variable for that sample. For a training set with M samples, LOO generates predicted values for all M-dependent variables via M iterations. In contrast, LMO divides the training set into consecutive subsets. In each iteration, one subset is excluded, and the model is developed with the remaining data to predict the dependent variable for the excluded subset, following a similar approach to LOO.

The model construction initially relies on the training set for development and internal validation. Metrics such as RMSEP and *R*^2^ are calculated by comparing experimental values with predicted values [15]. However, high predictivity from internal validation alone is insufficient to confirm the robust predictive capability of a model. External validation is typically considered the most reliable method for assessing the robustness and RT prediction accuracy of a model for analytes that are not included in the training set [93]. The primary objective of external validation is to establish a reliable model based on carefully selected descriptors rather than relationships formed by chance. Well-trained and validated models are essential to achieve strong predictive performance [94].

## 3 Application of QSRR

### 3.1 QSRR for compound identification

#### 3.1.1 Identification of small molecules

**Fig. 6:**
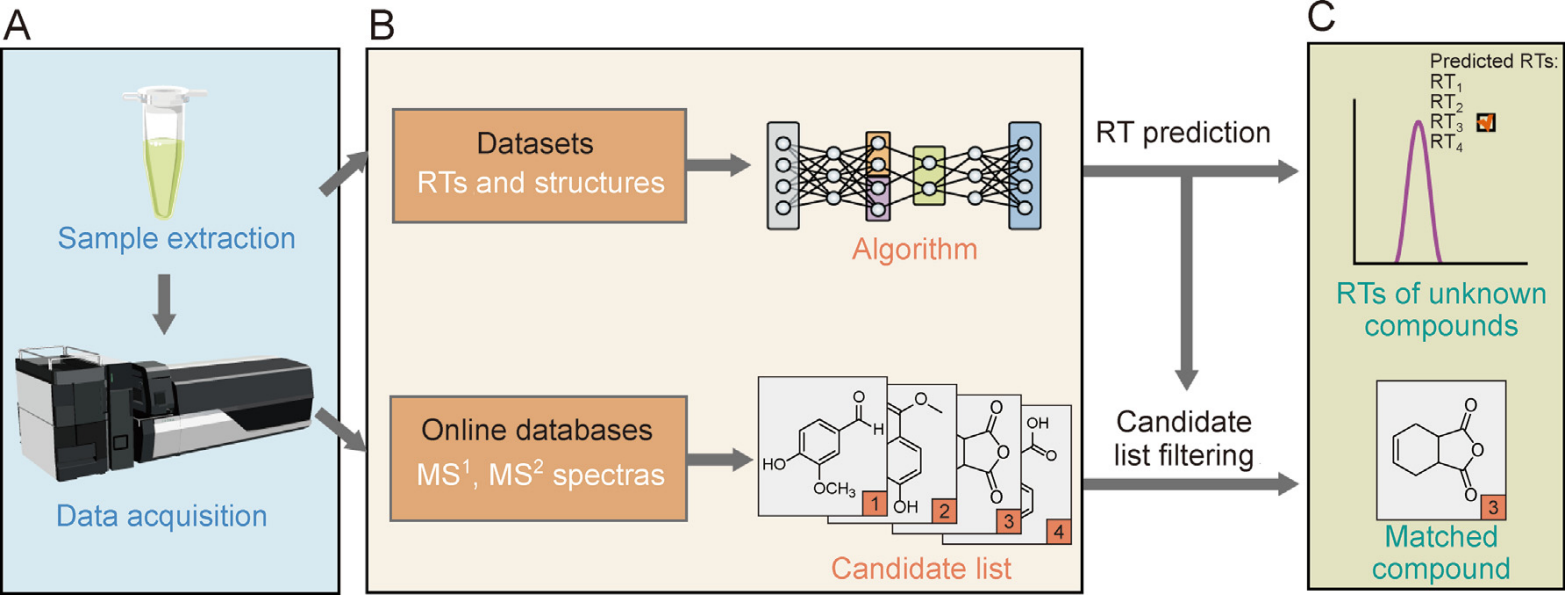
Schematic representation of the compound identification process using retention time (RT) prediction and mass spectrometry (MS) data. (A) Sample preparation and data acquisition. (B) Candidate generation and RT prediction: MS^1^ and MS^2^ information from online databases are matched to generate an initial candidate list, while quantitative structure-retention relationship (QSRR) models are applied to predict RT values. (C) Candidate filtering and identification: Predicted RTs are used to filter the candidate list, reducing false identification and ultimately identifying the matched compound by combining with MS^1^ and MS^2^ spectra.

Identifying compounds based solely on MS signals often leaves many compounds unidentified, which is commonly referred to as “dark matter” owing to the limitations of this approach [95]. RTs provide an additional dimension of information that, when combined with MS fragmentation patterns, significantly enhances identification accuracy and reduces misidentification rates [96]. This underscores the importance of accurate RT predictions, particularly when standards are unavailable [85]. For example, Wei et al. [97] constructed a QSRR model based on 43 steroid standards to predict RTs and summarized the fragmentation rules for derivatized carbonyl steroids, enabling the identification of 93 potential carbonyl steroids in human serum and demonstrating the effectiveness of RT predictions in compound identification. Similarly, Polettini et al. [98] developed a computational approach to identify synthetic cannabinoids by starting with the molecular formula of a suspected synthetic cannabinoid and searching for isomers across various new psychoactive substance databases. By integrating predicted RTs with MS data, this approach reduced the number of retrieved isomers by two-thirds (from 2792 ± 3358 to 845 ± 983), making it easier to distinguish isomers, particularly when MS spectra were nonselective or unavailable. Fig. 6 illustrates the process of RT prediction and its integration with MS data to enhance compound identification accuracy.

#### 3.1.2 Identification of peptides

RT information significantly enhances peptide identification accuracy when combined with MS data. Integrating predicted RTs with MS fragmentation data enables the creation of more comprehensive libraries and significantly improves peptide identification from data-independent acquisition data [99]. As highlighted in previous reviews, our understanding of the complex interactions between the physicochemical properties of peptides and the stationary phase is limited, which can result in suboptimal RT predictions for peptides [100]. However, the availability of large datasets presents opportunities to improve peptide RT prediction using advanced machine-learning techniques. In particular, deep learning is promising for addressing the challenges associated with peptide RT prediction.

For example, DeepRT, built using a capsule network model, demonstrated high accuracy of RT prediction with an *R*^2^ of 0.994 on a public dataset of peptides separated by RPLC, 0.993 for HILIC, and 0.996 for IC [101]. When large peptide datasets are available, DeepRT can be extended to DeepRT(+) via transfer learning. Similarly, DeepLC achieved an *R*^2^ > 0.99 across multiple datasets of unmodified peptides spanning different chromatographic systems [102]. Unlike previous models, DeepLC introduced novel peptide encoding based on atomic composition, enabling more accurate RT predictions for previously unseen, modified peptides. These advancements in RT prediction models have significantly reduced the ambiguity associated with peptide identification. Given their improved predictive power, these models have the potential to enhance ion libraries through in silico RT predictions and increase the peptide match rate between the ion library and data-independent acquisition data.

### 3.2 QSRR in the development of chromatographic methods

Method development is a dynamic and essential aspect of chromatography and is particularly important in pharmaceutical, drug analysis, bioanalysis, and medicinal chemistry. The primary objective is to identify optimal separation conditions for various analytes, including selecting an appropriate stationary phase and designing a suitable mobile phase, which may involve adjusting the concentration and type of organic modifiers, buffers, and pH. During method optimization, key parameters such as the selectivity factor (α) and resolution are considered critical quality attributes because they significantly influence the separation quality. Traditionally, method development has relied on trial-and-error experiments, involving the extensive testing of various chromatographic conditions such as mobile phase composition, stationary phase selection, temperature, and flow rate adjustments. QSRR provides a more efficient alternative, significantly reducing the experimental workload required for method development.

#### 3.2.1 Model-based column characterization

Chromatographic column characterization involves evaluating and describing the physicochemical properties of the stationary phase, which directly affects the RT and separation efficiency of the analytes. This characterization is crucial for optimizing the separation conditions and improving the understanding of the analyte behavior during chromatography. Various QSRR methods have been used for characterization [103], with the LSER model being the most common. The LSER model describes and predicts interactions between analytes (solutes) and the stationary or mobile phase (solvents), encompassing five key solute–solvent interactions: hydrogen bond acidity (*A*), hydrogen bond basicity (*B*), excess molar refraction (*E*), dipolarity/polarizability (*S*), and either the logarithm of the hexadecane-gas partition coefficient (*L*) or the characteristic McGowan volume (*V*). These parameters explain solute behavior in chromatography and predict their interactions with the stationary and mobile phases during separation. These interactions were modeled using the following equations:

(1) *SP* = a*A +* b*B +* s*S +* e*E +* l*L* + c

(2) *SP* = a*A +* b*B +* s*S +* e*E +* v*V +* c

In these equations, capital letters denote solute properties, and lowercase letters denote system constants. When LSER is applied to chromatography, the *SP* in Eqs. (1), (2)) represents retention. Eq. (1) is preferred for GC indices, whereas Eq. (2) is specific to LC retention data. The coefficients were determined using an MLR model to characterize the chromatographic system.

In 2021, Poole [104] demonstrated that the accurate characterization of a chromatographic system requires at least 35 compounds, making the process time-consuming. To address this issue, a faster characterization method using pairs of test compounds was proposed [105], drawing inspiration from Tanaka's scheme [106]. This method reduces the number of required compounds and accelerates the reaction. Furthermore, Schuster and Lindner [107] used a modified LSER [108] approach to characterize 22 HILIC columns using 68 probe compounds.

The QSRR methodology combined with HSM is effective for column characterization [54]. In HSM, analyte retention is modeled as a linear relationship between five column coefficients (*H*, *S*, *A*, *B*, and *C*) and five complementary solute coefficients (η′, σ′, β′, α′, and κ′) as follows:

(3) log*α* = log *(k/k*_*EB*_*)* = *η′*H − *σ′*S + *β′*A + *α′*B + *κ′*C

Where α represents chromatographic selectivity, k is the solute retention factor, and k_EB_ is the retention factor of ethylbenzene. However, the experimental determination of solute coefficients is time-consuming, limiting the applicability of HSM to RT prediction [109]. A promising solution is to predict solute coefficients using HSM. Based on the solvophobic theory [109], hydrophobic interactions are the primary contributors to the analyte retention in RPLC. Wen et al. [54] introduced an approximate HSM that focused solely on hydrophobicity:

(4) log*α* = log *(k/k*_*EB*_*)* = *η′*H

Wen et al. [58] developed a QSRR model using 49 compounds to predict η′ in the approximate HSM, which enabled RT prediction based on the provided column parameters. Six RP columns and two datasets under identical isocratic conditions were used to validate the QSRR model. The predicted resolution values were compared to identify the best-fitting column, and the actual chromatograms confirmed that column selection could be achieved from the predicted values without experimentation.

#### 3.2.2 Method optimization in chromatography

##### 3.2.2.1 Traditional QSRR methodology for method optimization

Traditional QSRR models use the relationship between RT and chemical structure to predict RTs for new analytes outside the training dataset. Calculating the α value from these predicted RTs enables the evaluation of the effectiveness of the chromatographic method in optimizing analyte separation and overall performance. However, because RTs vary with changes in chromatographic conditions, optimizing separations using the traditional QSRR methodology often requires multiple experiments, each conducted under different conditions. This process usually involves systematically changing one variable at a time while keeping other variables constant [110]. Consequently, traditional QSRR models must be recalibrated multiple times to identify the optimal separation conditions based on the predicted results, making this approach laborious and time-consuming.

##### 3.2.2.2 Model-based QSRR for method optimization

The model-based QSRR methodology provides an alternative to traditional trial-and-error experimentation, encompassing methods such as the design of experiments (DoE)-, HSM-, and linear solvent strength (LSS)-based QSRR. A quality-by-design framework is crucial for identifying optimal separation conditions because it systematically explores a preferred design space through DoE. Commonly used for developing RPLC methods, DoE is implemented in software such as Drylab [111,112]. This process involves selecting a stationary phase and defining a search area for the mobile phase composition, considering factors such as organic modifier content, pH levels, and ionic strength [113]. A statistical retention model is then constructed to relate the analyte RTs with these variables during model generation.

DoE-based QSRR predictions have been successfully applied in various studies. For example, Wiczling et al. [114] used a limited experimental design with a posteriori Bayesian method to optimize parameters, enabling the prediction of RTs of diverse compounds for different mobile phase ratio. In another study, Taraji et al. [115] combined QSRR with DoE to predict RTs for new analytes and different mobile phase ratio, which were used to calculate α, extending RT predictions to a broad experimental range.

In addition to DoE, HSM and LSS models have also been integrated with QSRR for the development of chromatographic methods. Wen et al. [54] combined VolSurf + descriptors with PLS and a GA to predict HSM coefficients and achieved high accuracy in assessing the likelihood of analyte coelution. This approach was validated using five analytes across nine RP columns, highlighting the potential of QSRR for identifying potential coelution issues during the initial stages of pharmaceutical analysis.

The LSS model correlated RTs with eluent concentration, particularly IC. The model uses the following equation:

(5) log *k* = a − blog *[eluent]*

**Fig. 7:**
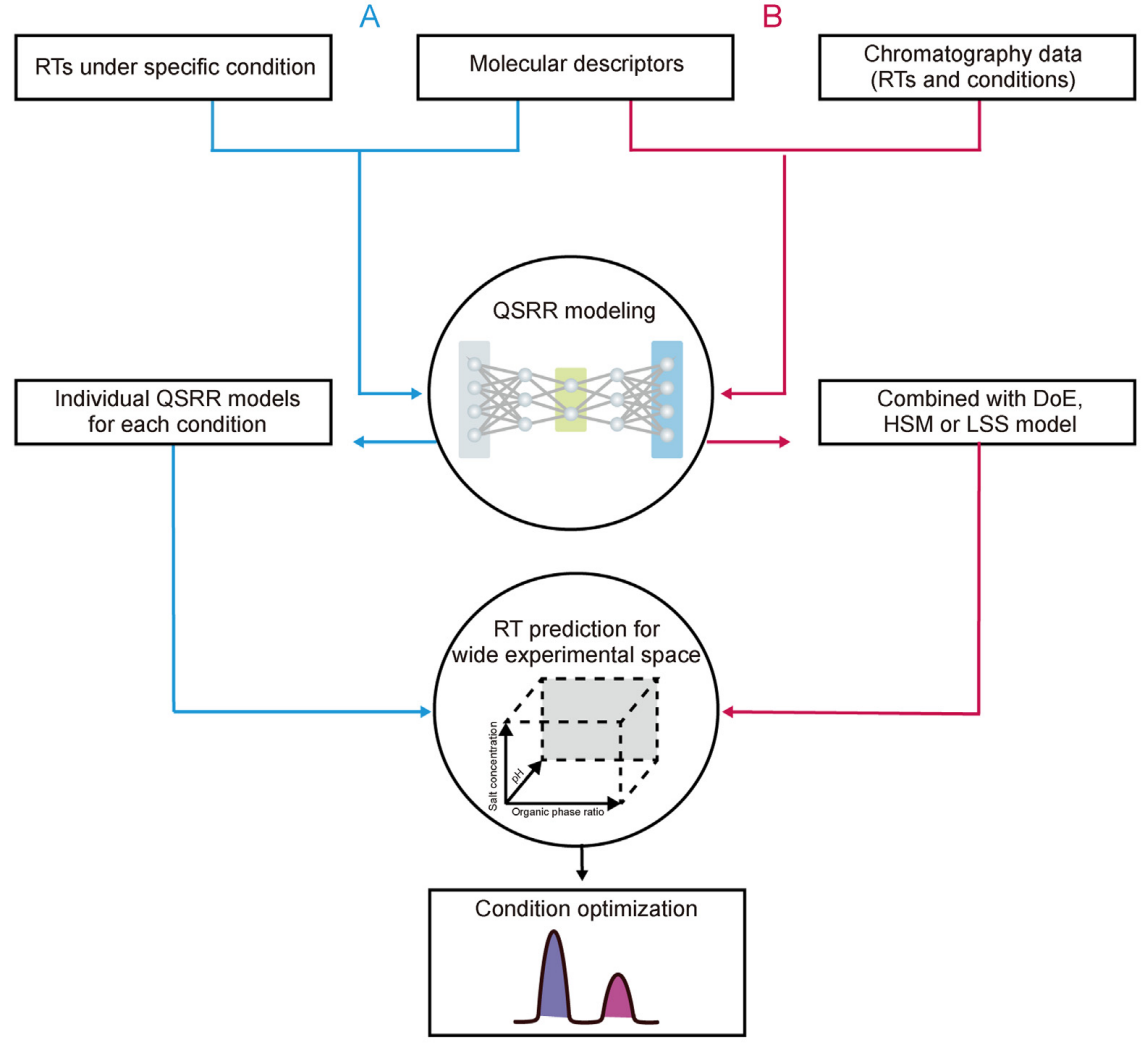
Illustration of two distinct quantitative structure-retention relationship (QSRR) concepts in chromatographic method development. (A) Traditional QSRR methodology: Individual QSRR models are built for each set of experimental conditions, predicting retention times (RTs) under specific conditions. These QSRR models can be used together to predict RTs across different experiment conditions, allowing for condition comparison and optimization. (B) Model-based QSRR methodology: QSRR models are combined with design of experiment (DoE), hydrophobic subtraction model (HSM), or linear solvent strength (LSS), enabling RT predictions across a broader experimental space and facilitating condition optimization.

Where [eluent] represents the eluent concentration, and a and b are the intercept and slope, respectively. By correlating these parameters with the solute structure, predicted values for a and b for any given solute can be inserted into the LSS model to estimate RTs at various eluent concentrations. Park et al. [116,117] demonstrated this approach by predicting a and b values using the LSS model and subsequently using these predictions to predict RTs across different eluent concentrations, thereby facilitating the optimization of chromatographic conditions. To elucidate the differences between the traditional and model-based QSRR methodologies, a visual comparison was performed, which is presented in Fig. 7.

### 3.3 QSRR in drug screening

In the early stages of drug discovery, evaluating the physicochemical properties of each drug candidate is critical for biological activity screening [118]. Immobilized artificial membrane (IAM) chromatography mimics the biological membrane environment, enabling researchers to assess drug interactions with membrane components such as phospholipids [119]. This technology plays a key role in drug screening by predicting blood–brain barrier permeability [120], absorption, and distribution, thereby supporting bioavailability and pharmacokinetics evaluations [[121], [122], [123]]. By early identification of compounds with favorable membrane interactions, IAM chromatography enhances the efficiency and success rate of drug development. In IAM chromatography, analyte retention can be measured via two methods: isocratic elution to determine logk_wIAM_ parameters and gradient elution to measure the IAM chromatographic hydrophobicity indices (CHI_IAM_) [124]. Ciura et al. [124] used IAM chromatography to simulate xenobiotic-phospholipid interactions and developed a QSRR model with ANN and MLR to predict CHI_IAM_ values. This model accurately predicted the molecular affinity of phospholipids, facilitating the rapid evaluation of drug interactions with biological membranes. In a subsequent study, Ciura et al. [125] performed QSRR analysis on the CHI_IAM_ values using differential evolution and PLS. This model revealed the effects of physicochemical properties on the retention behavior of isoxazolone derivatives in IAM chromatography, facilitating the assessment of the phospholipid affinity of newly synthesized isoxazolone derivatives and enabling comparisons with lipophilicity parameters from RPLC.

### 3.4 QSRR for analyzing retention mechanisms

The QSRR approach, which uses interpretable descriptors, is crucial for understanding the molecular mechanisms that influence chromatographic performance. By analyzing the effects of various molecular descriptors on retention across different stationary phases, QSRR can be used to gain valuable insights into solute behavior in diverse systems. Many studies on retention mechanisms in RPLC have highlighted the importance of descriptors such as hydrophobicity and hydrogen bonding in RT prediction [126,127], whereas other molecular size–independent intermolecular interactions are generally less influential [128]. QSRR models have been used to analyze the retention mechanisms of different analytes on different stationary phases, including alkylbenzenes on microdispersed sintered nanodiamonds and amino acids on 12 HILIC stationary phases [[129], [130], [131]]. For example, Kaczmarek et al. [132] developed a QSRR model to link molecular structure descriptors with the chromatographic retention parameter logk_w_, facilitating the analysis of retention mechanisms across different stationary phases. This study shows that these descriptors can effectively distinguish separation mechanisms, especially by highlighting analyte interactions between stationary and mobile phases (notably in acetonitrile-based systems).

## 4 Challenges and potential solutions to QSRR RT prediction

### 4.1 RT transfer across different chromatographic systems

#### 4.1.1 Predicting RT using proven models

A major challenge in QSRR is its limited applicability across diverse chromatography systems, as varying experimental setups result in RT shifts [133]. Incorporating data from various chromatographic systems can help the model learn and adapt to different experimental conditions and enhance its predictive accuracy; however, this approach faces practical challenges, including high costs, time constraints, data quality and diversity issues, increased model complexity, integration issues, and data sparsity. An alternative approach is to use the QSRR model to predict key parameters related to RT, which are then integrated into an established chromatographic model to indirectly predict RT. The most common QSRR models are the HSM- and LSS-based QSRR models. Studies have successfully applied this method to predict parameters within the HSM framework for RPLC, facilitating accurate RT prediction for different chromatographic columns [54,58,60]. For example, Wen et al. [58] used a QSRR model to predict HSM coefficients and achieved promising results with new C_18_ columns.

Additionally, researchers have integrated QSRR with LSS models to predict RTs in IC, enabling predictions across various elution concentrations, including gradient elution and multistep eluent conditions [57,134]. In these studies, QSRR predicted the regression parameters a and b for analytes not included in the model's generation. The predicted parameters were then incorporated into the LSS model to estimate the RT. Park et al. [116] constructed a QSRR model to predict the a and b parameters of new analytes. Using these parameters in the LSS model, highly accurate RT predictions were obtained, with an *R*^2^ of 0.98 and an RMSEP of 0.89 min.

#### 4.1.2 RT projection

Chromatographic analysis is inherently complex because of the various analytical instruments, gradients, and columns used [135]. Transferring RTs between different laboratory settings or chromatographic conditions often requires rebuilding QSRR models, which can be impractical. An alternative solution is RT projection, which establishes a functional relationship between existing and target chromatographic systems.

Studies have demonstrated that RT projections from isocratic to gradient elution can achieve high accuracy [[136], [137], [138]]. Recent improvements in gradient-to-gradient elution projection methods have minimized error although they require validation against a subset of standards [136,137]. Tools such as PredRet [22] enable direct RT projections across multiple chromatographic systems with superior accuracy compared to the development of new QSRR models.

Most RT projection methods rely on an “experimental–experimental projection” approach, in which RTs from one system are projected onto another using experimental data from both systems. This approach achieves high accuracy with minimal error when there is sufficient overlap of compounds between the two systems. To address this limitation, one solution is to use predicted values for mapping. Domingo-Almenara [14] used “predicted-experimental projections”, which provide a projection function that enables predicted RTs to be projected onto a new chromatographic system. Although “predicted-experimental projections” typically exhibit higher error than “experimental–experimental projections”, they perform better than models generated from small datasets under specific conditions [83].

Another approach involves meta-learning projections. García et al. [86] demonstrated that meta-learning can generate a projection function using 10 identified metabolites, revealing that the effectiveness of these projection functions is highly dependent on training inputs. Although this method is promising, it is limited by its reliance on initial dataset overlap, highlighting the need for new strategies to predict RTs beyond the scope of the original dataset.

#### 4.1.3 Transfer learning

Transfer learning is a deep learning technique that offers promising solutions to the challenges of transferring RTs between chromatographic systems [139]. By leveraging the feature extraction capabilities of a pretrained model on a large dataset, transfer learning enhances RT prediction performance in new chromatographic systems [140]. The effectiveness of this method has been demonstrated in similar types of chromatographic systems (such as RPLC to RPLC or HILIC). For example, in RPLC systems, a GNN model [80] was adapted for various systems by freezing the feature extraction layer of the METLIN SMRT-based pretrained model and training only the output layer, resulting in reliable predictions across 11 RPLC datasets. In the HILIC systems, Yang et al. [141] used the Retip and MoNA datasets for pretraining, achieving an 18% improvement in test set performance and a 32% increase in external validation performance compared to the original GNN model. Transfer learning has also been applied to different chromatographic systems, such as between RPLC and HILIC. For example, Ju et al. [85] pretrained the DNNpwa model on the METLIN SMRT dataset to analyze 17 datasets from various chromatographic systems. The results indicated that the transferred DNNpwa model outperformed RF, GBT, and DNN in most of the 17 datasets, including RPLC and HILIC data, demonstrating its ability to generalize effectively across different chromatographic systems.

### 4.2 RT prediction on small datasets

Developing QSRR models on small datasets poses significant challenges, primarily owing to the risks of overfitting and limited applicability arising from insufficient data coverage [142]. To address these issues, researchers have employed transfer learning and meta-learning. Transfer learning uses knowledge gained from large datasets to improve predictions in systems with limited data. For example, Wang et al. [142] integrated molecular representations with adaptive networks and applied transfer learning to improve RT predictions across 14 small datasets, effectively bridging data gaps and improving prediction accuracy despite the scarcity of available data.

Meta-learning has emerged as a promising solution for addressing the challenges associated with small datasets by learning from a series of meta-tasks and applying this knowledge to related target tasks [143]. García et al. [86] introduced a Bayesian meta-learning approach using the SMRT dataset to project RTs onto other chromatographic systems. Bayesian methods are particularly useful for small data regression problems because they incorporate prior knowledge and provide effective solutions with minimal sample size. A key challenge is to define an appropriate prior that is learned from meta-tasks and subsequently refined with new evidence from the target task, resulting in a posterior distribution. This method demonstrates significant potential for RT prediction, indicating that meta-learning can be increasingly used in future research.

### 4.3 Retention prediction for stereoisomers

Stereoisomers that interact stereoselectively with biological macromolecules can exhibit significantly different pharmacological properties despite their structural similarities [144]. Therefore, accurate RT predictions for stereoisomers are essential. However, most existing models fail to consider the 3D geometry of molecules, limiting their ability to distinguish between stereoisomers.

#### 4.3.1 Incorporating 3D descriptors

Most models overlook the 3D geometric information of molecules, which limits the accuracy of feature learning and extraction. However, several studies have demonstrated that incorporating geometric information significantly improves model performance. For example, Nitta and Kaneko [145] used Dragon software to calculate 3D descriptors and achieved highly accurate predictions of analyte activities. Bahia et al. [146] constructed three QSRR models based on 2D, 3D, and a combination of 2D and 3D descriptors, demonstrating that the combined model provided the most substantial improvement in accuracy. Randazzo et al. [147] developed QSRR models using VolSurf+ 3D molecular descriptors along with a novel gonane topological weight fingerprint, effectively characterizing chiral centers in steroid homologs and enhancing model performance under various chromatographic conditions.

#### 4.3.2 Exploring 2D and hybrid methods

Owing to the time-consuming nature of simulating molecular 3D conformers for 3D descriptor calculations, researchers have begun exploring alternative methods. One such method involves using 2D image-based QSRR models. Barfeii and Garkani-Nejad [148] used multivariate image analysis descriptors to construct a QSRR model that demonstrated better differentiation between (R) and (S) isomers than traditional structural methods. In addition, some innovative 2D structure-based QSRR models have integrated selective 3D features, such as generating 3D molecular coordinates from 2D structures and using atom distance-based and 2D fingerprint-based 3D molecular fingerprints [149]. Although these methods have shown improved performance in differentiating stereoisomers compared to 2D-based strategies, they still fall short because of the absence of complete 3D information.

#### 4.3.3 Advanced 3D models

Recent advancements, such as DimeNet and SphereNet, have attempted to capture 3D information, including the Euclidean distances between atoms, bond angles, and torsion angles, resulting in improved prediction accuracy. However, these models require more comprehensive data to effectively correlate conformers with RTs. For example, Du et al. [150] adopted a chemical feature fusion network that integrates 2D structural information with 3D geometry. This model successfully distinguished configurational, stereoisomeric, and conformational isomers, achieving excellent prediction performance with an average error of 0.0143 for a dataset of 1,500 chiral conformers.

## 5 Conclusions and outlook

QSRR is a prominent research field in chromatographic analysis and is crucial for predicting RTs by linking the molecular structure of a compound to its retention behavior. This review provides a comprehensive overview of the QSRR workflow, encompassing retention database collection, feature calculation and selection, dataset partitioning, regression model development, model evaluation, and QSRR applications, with a particular emphasis on the role of AI. QSRR is used in various fields, including compound identification, chromatographic method development, drug screening, and retention mechanism analysis. In compound identification, predicted RTs serve as an orthogonal strategy that complements molecular mass and fragment ion data. This approach effectively eliminates many false-positive candidate structures, particularly for isomers with identical masses, thereby enhancing the accuracy and reliability of identification. For method development and optimization, analytical techniques based on systematic RT prediction are more efficient and robust and significantly decrease the time required for experimental design compared to traditional trial-and-error methods. For drug screening, QSRR establishes a quantitative relationship between the chemical structure of a compound and its RT, enabling predictions of retention behavior in biochromatography systems. By predicting the RTs of numerous candidate compounds, researchers can prioritize compounds with favorable retention profiles for further experimental validation, improving screening efficiency. Furthermore, QSRR identified key factors that influence retention, providing deeper insights into the mechanism underlying retention.

The rapid advancements in large language models and generative AI over the past two years have significantly enhanced the capabilities and impact of AI. Consequently, QSRR modeling based on physicochemical properties has become significantly accessible, even for individuals without a computer science background. However, predicting RT is challenging in three key areas: transferring RTs across different chromatographic systems, working with small datasets, and predicting RTs for stereoisomers. The complexity of experimental conditions often complicates the application of the same QSRR model to different laboratories or experimental setups. This has prompted the development of strategies, such as integrating QSRR with established chromatographic models, RT projection, and transfer learning. In the case of small datasets, a lack of sufficient experimental data can lead to overfitting, which can be mitigated using transfer learning and meta-learning methods. Lastly, predicting RTs for stereoisomers is hindered by the limitations of current descriptors and models, which fail to adequately capture the 3D information necessary for effectively distinguishing stereoisomers. To address this issue, incorporating 3D descriptors and developing advanced 3D models have been proposed as potential solutions for the better utilization of structural information. This review provides a comprehensive understanding of QSRR methodology, highlighting its current challenges and potential for future advancements in AI-driven chromatography, thereby making a valuable contribution to the field of analytical chemistry.

## CRediT authorship contribution statement

**Jingru Xie:** Writing – original draft. **Si Chen:** Writing – review & editing. **Liang Zhao:** Supervision. **Xin Dong:** Project administration, Conceptualization.

## Declaration of generative AI and AI-assisted technologies in the writing process

During the preparation of this work, the author(s) used ChatGPT to polish the anguage. After using this tool/service, the authors reviewed and edited the content as needed and take full responsibility for the content of the publication.

## Declaration of competing interest

The authors declare that there are no conflicts of interest.
